# Immunomodulatory Role of Propolis in Hypoxia and in the Tumor Microenvironment

**DOI:** 10.3390/molecules30224460

**Published:** 2025-11-19

**Authors:** Małgorzata Kłósek, Anna Kurek-Górecka, Radosław Balwierz, Katarzyna Góralczyk-Bałys, Michał Górecki, Zenon P. Czuba

**Affiliations:** 1 Department of Microbiology and Immunology, Faculty of Medical Sciences, Medical University of Silesia in Katowice, Jordana 19, 41-808 Zabrze, Poland; zczuba@sum.edu.pl; 2 Polish Apitherapy Society, ul. Jana Nowaka-Jeziorańskiego 9/u6, 03-984 Warsaw, Poland; radoslaw.balwierz@uni.opole.pl (R.B.); kgoralczyk@op.pl (K.G.-B.); mgorecki@sum.edu.pl (M.G.); 3 Institute of Chemistry, University of Opole, Oleska 48, 45-052 Opole, Poland; 4 Department of Drug and Cosmetics Technology, Faculty of Pharmaceutical Sciences in Sosnowiec, Medical University of Silesia in Katowice, St. Jednosci 8, 41-200 Sosnowiec, Poland

**Keywords:** propolis, hypoxia, cytokines, tumor microenvironment

## Abstract

Propolis is a well-known sticky, resinous substance collected by honeybees (*Apis mellifera*) from the buds of trees and other plants, then mixed with beeswax and their own glandular secretions. Its chemical composition varies widely depending on the bee species, geographic location, plant sources, and weather conditions. The therapeutic potential of propolis—including antimicrobial, anti-inflammatory, and anticancer effects—has been recognized since antiquity. Cancer remains one of the leading causes of morbidity and mortality worldwide. High levels of hypoxia within tumor tissue significantly contribute to cancer progression and increase the resistance of tumor cells to radio- and chemotherapy. In the tumor microenvironment, cytokines play key roles in processes such as invasion, metastasis, and immune suppression. The concept of the “cytokine field” describes how elevated cytokine levels within the tumor microenvironment create a field effect, influencing surrounding cells. Current research is exploring the use of natural immunomodulators, such as propolis, in combination with conventional chemotherapeutic agents for cancer treatment. This review summarizes the potential immunomodulatory role of propolis within the tumor microenvironment.

## 1. Introduction

Propolis (bee glue) is a resinous mixture produced by honey bees, made up of wax and partially digested plant buds and leaves. Bees use it to coat their hives, protecting them from microorganisms and predators. It also plays a structural role: sealing gaps, stiffening the combs for stability, preventing water from entering, and helping maintain a constant internal temperature of about 35 °C. Additionally, bees use propolis to encase and preserve the body of any intruder that dies inside the hive, preventing its decomposition [1]. Propolis has been employed in medicinal practices since antiquity. Iconographic evidence, such as depictions of bees on ancient Egyptian vases, suggests its early cultural significance. Historical sources indicate that the therapeutic properties of propolis were recognized in ancient Greece, Rome, Persia, and the Arab world, where it was applied primarily for wound treatment and as an antiseptic agent. In Egypt, propolis was additionally utilized in the process of embalming, underscoring its role in both medical and ritual contexts [2].

Propolis exhibits considerable variability in color (brown, yellow, green, or red) and odor depending on its botanical and geographical origin [3]. To date, more than 300 compounds have been identified, including phenolic acids, flavonoids, terpenoids, ketones, aromatic acids and esters, aliphatic acids, aldehydes, fatty acids, carbohydrates, amino acids, minerals, and vitamins [4]. The chemical composition of propolis is influenced by such factors as honeybee species, local flora, and seasonal conditions. Propolis is widely recognized for its diverse biological activities, including antibacterial, antifungal, antiparasitic, antiviral, anti-inflammatory, antioxidant, and wound-healing properties [5].

To fully appreciate the therapeutic potential of propolis introduced above, it is essential to first understand the foundation of its bioactivity: its complex and highly variable chemical composition. The diverse array of compounds found within propolis from different geographical regions, which will be detailed next, directly dictates its pharmacological effects.

In Europe, North America, non-tropical regions of Asia, and New Zealand, propolis is derived primarily from the buds of black poplar (*Populus nigra*) [1]. Although its chemical profile is heterogeneous and strictly dependent on geographical and botanical origin, the potent biological activity of propolis, including its anticancer effects, is primarily attributed to a high content of polyphenolic compounds. In European-type propolis, predominantly sourced from poplar trees (*Populus* sp.), the key bioactive constituents are flavonoids (e.g., chrysin, pinocembrin, galangin) and phenolic acid esters, with Caffeic Acid Phenethyl Ester (CAPE) being the most prominent agent [6,7,8,9].

Conversely, for the extensively studied Brazilian propolis, the green type sourced from *Baccharis dracunculifolia* is distinguished by its main active component, artepillin C, a prenylated derivative of *p*-coumaric acid. These specific molecules—CAPE and artepillin C—exert potent, pleiotropic effects, including anti-proliferative, pro-apoptotic, anti-angiogenic, and immunomodulatory activities, positioning them as central to the analysis of the tumor microenvironment [6,10,11]. This review will now focus on analyzing the mechanisms of action positioning CAPE and artepillin C as pleiotropic molecular agents that directly interfere with the central HIF-1α, NF-κB, and STAT3 signaling pathways, thus breaking down the hypoxic and inflammatory structure of the TME. These compounds will form the analytical basis for further discussion.

## 2. Methods

Although the literature provides extensive information on the activity of propolis in various cancer types, data on the immunomodulatory role of propolis in hypoxia and in the tumor microenvironment remain relatively scarce. This article is based on a structured literature review. This approach aimed to provide an objective and comprehensive assessment of the available scientific evidence. To this end, a systematic search was conducted across the PubMed, Scopus, Web of Science, and Google Scholar databases for publications from 1 July until 5 November 2025. The search, reading, review and collection process was conducted by Małgorzata Kłósek and Anna Kurek-Górecka. The selection criteria for the papers included and excluded in this review are presented in Table 1.

This article is a structured narrative review, for which the search and selection process was guided by the principles of the Preferred Reporting Items for Systematic Reviews and PRISMA statement. A systematic search was conducted across the PubMed, Scopus, and Web of Science databases for publications up to October 2025, with no language restriction initially applied, although the final analysis was limited to English-language articles. The search strategy combined MeSH terms and keywords, including: (“propolis” OR “bee glue”) AND (“cancer” OR “tumor” OR “carcinoma”) AND (“hypoxia” OR “HIF-1alpha”) AND (“microenvironment” OR “TME” OR “cytokine” OR “inflammation” OR “NF-kB” OR “STAT3”). The reference lists of retrieved articles were also manually screened to identify additional relevant publications.

A total of 1247 records were initially identified. After removing 312 duplicates, 935 records were screened based on title and abstract, which resulted in the exclusion of 801 irrelevant studies. The full texts of the remaining 134 articles were assessed for eligibility. Of these, 92 articles were excluded for not meeting the inclusion criteria (e.g., lacking specific mechanistic data relevant to the review’s focus). A final total of 42 studies were included in the narrative synthesis, forming the core evidence base for this review. The final manuscript utilizes 160 references, which include these 42 core studies plus additional foundational and contextual citations.

The study selection was performed in a two-step process by two independent reviewers (M.K., A.K.-G.) to ensure objectivity and rigor. In the first step, titles and abstracts of all retrieved articles were screened for relevance based on the pre-defined inclusion criteria. In the second step, the full texts of the potentially eligible articles were thoroughly assessed. This dual-reviewer approach was implemented to minimize selection bias; any discrepancies regarding the inclusion of specific studies were resolved through discussion and consensus to ensure adherence to the pre-defined criteria. Given that the objective of this review was a narrative synthesis of mechanistic evidence rather than a quantitative meta-analysis, a formal PRISMA flow diagram was not generated. Data regarding research models, propolis origin and composition, its effects on signaling pathways, and cytokine expression profiles were systematically extracted from the final selection of articles to construct a coherent and evidence-based narrative.

Having established the chemical arsenal of propolis, the discussion now turns to the complex biological context in which these compounds exert their anti-cancer effects: carcinogenesis. Understanding the multi-stage nature of tumor development is essential for identifying the specific pathways that propolis may target.

## 3. Carcinogenesis and the Role of Hypoxia in This Process

In the multi-stage process of carcinogenesis, a critical turning point is the development of hypoxia, leading to the activation of the HIF-1α signaling pathway [12]. This pathway not only drives tumor adaptation and survival but also represents a primary molecular target for the bioactive constituents found in propolis, as will be detailed later in this review. Carcinogenesis is a multistage process driven by genetic and epigenetic alterations culminating in angiogenesis and metastasis [13,14]. This process is frequently initiated and sustained by chronic inflammation, where inflammatory mediators disrupt cellular signaling to promote tumor initiation, invasion, and growth [15,16,17].

Hypoxia, a prominent feature of the solid tumor microenvironment, promotes therapeutic resistance and metastasis. It forces cancer cells to undergo metabolic reprogramming towards increased glycolysis and de novo lipid synthesis to support survival and proliferation [18]. This metabolic shift is regulated by hypoxia-inducible factor 1 (HIF-1α), which activates the transcription of key metabolic genes and reprograms oxidative metabolism to support continued proliferation under low-oxygen conditions.HIF-1α activation regulates this metabolic shift by inducing the transcription of key glycolytic enzymes and glucose transporters (e.g., GLUT1, GLUT3), thereby enhancing glucose uptake and glycolysis to support proliferation under low-oxygen conditions [19,20,21].

Crucially, hypoxia is a potent trigger for inflammation. The HIF pathway directly stimulates the expression of pro-inflammatory cytokines and modulates the function of various immune cells, thereby establishing a self-reinforcing loop where hypoxia drives inflammation and vice versa [22,23].

HIF-1α has been reported to elevate levels of TNF-α, IL-1β, IL-6 and CXCL8 (IL-8), while HIF-2α increases TNF-α (Tumor Necrosis Factor alpha), IL-1β, IL-6 and IL-12 [24,25]. In addition, HIF-1α participates in the activation of vascular endothelial growth factor (VEGF) transcription, a key stimulator of angiogenesis [21]. In particular, TNF-α and IL-1β contribute to regulatory of HIF pathway [26]. TNF-α modulates the sensitivity of the HIF pathway to hypoxia in inflamed cells and regulates protective HIF responses. It controls HIF-1α transcription via the NF-_K_B pathway. Chronic inflammation leads to the sustained release of pro-inflammatory cytokines such as TNF-α, IL-1β, and IL-6, together with chemokines, which collectively enhance cancer cell survival, proliferation, angiogenesis, and invasion. IL-1β, under normoxic condition, induces HIF-1α expression and modulates it post-transcriptionally, enhancing HIF-1α functional response in cells. Chronic inflammation has been shown to promote metaplasia, dysplasia, and neoplasia. Therefore, the tumor microenvironment significantly impacts cancer growth, progression, invasion, and metastasis [27,28].

The cellular response to hypoxia is largely arranged by a complex network of signaling molecules that mediate communication within the tumor microenvironment. Central to this network are cytokines, which not only drive inflammation but are also intricately linked to the hypoxic response, collectively creating a self-reinforcing loop that promotes tumor invasion, angiogenesis, and immune suppression.

Pro-inflammatory cytokines play a central role in tumor development and progression by modulating the tumor microenvironment, regulating immune responses, and influencing angiogenesis, lymphangiogenesis, and metastasis. Cytokines are proteins typically under 30 kDa that mediate communication between immune cells and coordinate interactions with other cells in the microenvironment [29]. Upon binding to receptors on target cells, cytokines activate intracellular signaling pathways that regulate gene transcription and, consequently, modulate diverse biological processes [30]. Receptors can also be shed from the cell surface, and some soluble forms retain the ability to bind ligands. The formation of such complexes can either inhibit or enhance cytokine activity. Cytokines are classified into distinct superfamilies based on their structural similarity and shared receptor subunits. These include interleukins (ILs), interferons (IFNs), TNFs, transforming growth factors (TGFs), colony-stimulating factors (CSFs), and chemokines (chemotactic cytokines) [31]. Common classifications also reflect their canonical activities, including pro-inflammatory, pro-angiogenic, inhibitory, hematopoietic, or chemotactic roles.

A core group of pro-inflammatory cytokines promotes this pro-tumorigenic environment. Key mediators such as IL-1β and TNF-α act as endogenous tumor promoters, driving angiogenesis, invasion, and metastasis. A pivotal player in this network is IL-6, which activates the potent pro-oncogenic JAK/STAT3 pathway, a process linked to the expansion of cancer stem cells and therapeutic resistance [32,33,34,35]. This cytokine cascade is complemented by chemokines like MCP-1 (CCL2), which recruit immunosuppressive cells to the TME, further sustaining the malignant phenotype [36,37,38].

Chemokines monocyte chemotactic protein-1 (MCP-1), known as C-C motif chemokine ligand 2 (CCL2), and Interferon gamma-induced protein 10 (IP-10), also known as C-X-C motif chemokine ligand 10 (CXCL10), play important roles in the development of tumors. MCP-1 is a potent chemoattractant involved in macrophage recruitment and the initiation of inflammation, thereby affecting tumor progression. Increased expression of MCP-1 in the tumor microenvironment is associated with invasion, metastasis, angiogenesis, and immune cell infiltration [39]. IL-1β has been shown to induce MCP-1 expression, with NF-κB and activator protein 1 (AP-1) mediating its regulation in renal cell carcinoma and glioblastoma multiforme [40]. In breast cancer, elevated concentrations of MCP-1 correlate with macrophage accumulation and angiogenesis [41].

IP10 binds to the CXCR3 receptor and it is involved in cancer pathogenesis by inducing apoptosis and regulating cell growth [42]. CXC chemokines exert a dual effect on angiogenesis, depending on the presence of the Glu-Leu-Arg (ELR) motif. The ELPR determines whether a chemokine has pro-angiogenic or angiostatic activity. Specifically, CXCL10, which lacks the ELR motif, functions as an angiostatic chemokine, inhibiting endothelial cell proliferation and thereby exhibiting anti-tumor activity [43,44].

Malignant tumors are characterized by rapid growth, resulting from intense cell division. However, as the tumor develops, cells increasingly experience oxygen deficiency due to the imbalance between the growing demand for oxygen and the limited supply. This deficiency arises from an insufficient number of blood vessels supplying the tumor. To overcome these unfavorable conditions and increase oxygen availability, angiogenesis is required. In addition, in order to maintain the highest possible levels of ATP (adenosine triphosphate), tumor cells adapt to these adverse conditions by increasing glucose transport and glycolysis. As early as the 1980s, Otto Warburg observed that cancerous tissues rely on a system in which glucose is converted to lactate for ATP synthesis, bypassing mitochondrial oxidative phosphorylation [45,46,47]. This shift in glucose metabolism is driven by the activation of HIF-1α under hypoxic conditions, which induces the transcription of genes involved in glucose uptake. Enhanced glycolysis in cancer cells not only supports energy production in low-oxygen environments but also provides essential precursors for the synthesis of nucleic acids, amino acids, and phospholipids required for cell proliferation under nutrient-limited conditions [20]. Tumor growth and metastasis depend on angiogenesis and lymphangiogenesis, which are induced by chemosignaling from rapidly proliferating tumor cells [48].

Angiogenesis plays a crucial role in carcinogenesis because it provides the oxygen and nutrients necessary for tumor growth, survival and metastasis. Cancer cells produce factors that stimulate the formation of new blood vessels. Tumor angiogenesis occurs in four stages. In the first stage, the basement membrane in tissues undergoes local damage, a process in which hypoxia plays a key role. In the second stage, endothelial cells activated by angiogenic factors begin to migrate. In the third stage, endothelial cells proliferate and stabilize, while in the fourth stage, angiogenesis is further regulated by angiogenic factors. Key angiogenic activators include vascular endothelial growth factor (VEGF), basic fibroblast growth factor (bFGF), angiogenin, transforming growth factor (TGF-α, TGF-β), tumor necrosis factor (TNF)-α, platelet-derived endothelial growth factor, granulocyte colony-stimulating factor, placental growth factor, interleukin-8, hepatocyte growth factor, and epidermal growth factor. Among these, VEGF is particularly important, acting as a mitogenic factor for endothelial cells and stimulating their proliferation.

While carcinogenesis describes the overall progression of cancer, a critical feature of the resulting solid tumor microenvironment is the development of hypoxic regions. This state of low oxygen is not merely a by-product of tumor growth but a fundamental driver of malignancy and therapeutic resistance, making it a pivotal aspect of the problem at hand. In summary, the hypoxic response coordinated by HIF-1α is the foundational event that establishes a pro-malignant landscape. However, its most critical downstream consequence is the potent and direct initiation of a chronic inflammatory state. HIF-1α acts as a master switch that not only triggers angiogenesis but also directly induces the transcription of key pro-inflammatory cytokines. This establishes an inextricable link between the low-oxygen environment and the development of a pro-tumorigenic immune milieu. The following section will therefore analyze this second, interconnected pillar of the TME, dissecting the cytokine network and the signaling pathways that are activated as a direct result of the hypoxic conditions previously described.

## 4. The Role of Cytokines in Tumor Microenvironment

Building upon the hypoxic foundation, the chronic inflammatory state within the TME is regulated by a network of cytokines. This response is governed primarily by the intracellular NF-κB and STAT3 signaling pathways, which drive the sustained production of key effector cytokines like IL-6 and TNF-α, thereby promoting immune evasion and therapy resistance. This inflammatory pathway constitutes the second critical therapeutic target [49,50,51,52]. TME is a dynamic ecosystem of immune and stromal cells (e.g., fibroblasts, tumor-associated macrophages) that regulates tumor progression via cytokine secretion. These mediators drive key pro-malignant processes, including angiogenesis, invasion, and cancer stem cell maintenance, while remodeling the extracellular matrix to create a therapy-resistant, hypoxic milieu [53]. The key cellular components and their secreted cytokines are summarized in Table 2. Cytokines secreted by immune cells within the tumor microenvironment, as well as by certain normal cells, play a crucial role in tumor progression and immune response. They promote various cancer-related processes, including angiogenesis, epithelial–mesenchymal transition (EMT), invasion, and the maintenance of cancer stem cells (CSCs) [54]. The structure of cancerous tumors exhibits features resembling tissue repair, characterized by an altered fibroblast phenotype, remodeling of the extracellular matrix, infiltration by reactive cells, increased protease activity, and an excess of biologically active cytokines. The ECM not only provides structural scaffolding but also drives malignant progression by releasing cytokines that sustain proliferation, suppress immune surveillance, and diminish chemotherapeutic efficacy [52]. Furthermore, hypoxia represents a key TME alteration that underlies tumor metabolism and progression.

Cancer cells are characterized by their ability to sustain continuous growth signals and by their capacity to synthesize and secrete numerous cytokines. They often express surface receptors, including cytokine receptors that are absent on their cells of origin, while receptors normally expressed at low levels on healthy cells are frequently upregulated. Cytokines play a central role in inflammation and specifically influence cellular communication and interactions. They can act in an autocrine manner on the secreting cells, in a paracrine manner on nearby cells, or in an endocrine manner on distant cells [55]. In many cancers, both lymphoid and non-lymphoid, autocrine cytokine signaling promotes tumor cell proliferation. Fibroblasts, the main non-immune stromal cells in the tumor microenvironment, not only produce and remodel the extracellular matrix but also promote cancer cell proliferation through the paracrine release of growth factors [56].

In this review, we focus on cytokines with established roles in human cancers. Individual cytokines may exert diverse, and sometimes opposing, effects depending on their cellular source, the differentiation state of target cells, and the surrounding microenvironment. Collectively, they form an interdependent network in which the production of one cytokine can stimulate or inhibit the production of, and responses to, others.

Within the TME, cellular populations such as cancer-associated fibroblasts (CAFs), M2-polarized tumor-associated macrophages (TAMs), and N2-polarized tumor-associated neutrophils (TANs) establish a complex pro-tumorigenic network. CAFs drive proliferation and vascularization through the secretion of growth factors and chemokines. M2-polarized TAMs and TANs contribute to angiogenesis, matrix remodeling, and immune suppression, fostering an environment conducive to tumor progression and metastasis [57,58,59,60,61,62,63,64,65,66]. Concurrently, other cells, such as Th2 and Th17 lymphocytes, sustain chronic inflammation, while the effector function of natural killer (NK) cells is profoundly impaired. This complex cellular crosstalk is also responsible for the generation of neutrophil extracellular traps (NETs), which can physically entrap cancer cells and promote their growth [67,68,69,70]. Excessive NETosis is also associated with chronic inflammation and tissue damage. Chronic inflammation, in turn, can lead to increased expression of adhesion molecules, which facilitates the immobilization of cancer cells and their interaction with the extracellular matrix (ECM).

Hypoxia also rapidly induces the upregulation of Intercellular Adhesion Molecule (ICAM-1) expression in inflamed tissues, particularly within the endothelium, via activation of HIF-1α. Elevated ICAM-1 levels substantially facilitate the directed migration of mesenchymal stem cells (MSCs) toward inflammatory sites. Moreover, hypoxia enhances the paracrine functions of MSCs, resulting in increased secretion of chemotactic and angiogenic mediators. MSCs stimulated with IFN-γ together with TNF-α or IL-1 display enhanced immunosuppressive capacity through upregulation of ICAM-1 and vascular cell adhesion molecule-1 (VCAM-1), both in vitro and in vivo. The inflammatory microenvironment is a critical determinant of MSC regulatory function. Under inflammatory conditions, M1 macrophages and Th1 lymphocytes release high levels of proinflammatory cytokines, endowing MSCs with pronounced immunomodulatory properties [71].

Table 2 shows the most important cells in the tumor microenvironment and the cytokines they secrete. In summary, the interplay between hypoxia (governed by HIF-1α) and chronic inflammation (driven by NF-κB and STAT3) creates a self-reinforcing network that fosters tumor malignancy. Having established this complex pathological landscape, the following section will now present a detailed analysis of the scientific evidence demonstrating how propolis and its bioactive compounds intervene at multiple levels, disrupting these core signaling pathways and thereby exerting their anticancer effects.

**Table 2:** Key cells and secreted by them cytokines in the tumor microenvironment.

Cell Type	Key Secreted Mediators	References
T helper 2 (Th2) cells	IL-1β, IL-4, IL-5, IL-6, IL-10, IL-13	[72]
B cells	IL-10, TGF-β	[73,74,75]
Tumor-associated neutrophils (TANs, N2-like)	IL-1β, IL-6, IL-17, TNF-α, CCL4, CXCL8, TGF-β, VEGF, MMP-9, Arginase-1 (ARG1)	[76]
Natural killer (NK) cells)	IFN-γ, TNF-α	[77]
Dendritic cells (DCs)	IL-6, IL-10, IDO1, ICOS-L, TGF-β	[78]
Tumor-associated macrophages (TAMs, M2-like)	IL-4, IL-6, IL-10, IL-12, TNF-α, TGF-β, VEGF, MMP-7, MMP-9, CCL2, CCL5, CCL-18	[60,79]
Cancer-associated fibroblasts (CAFs)	IL-6, IL-8, IL-11, IL-13, TGF-β, CXCL12, CXCL14, VEGF	[80]
Myeloid-derived suppressor cells (MDSCs)	IL-10, TGF-β, ARG1, IDO1, Nitric oxide (NO), Reactive oxygen species (ROS)	[78]
Cancer stem cells (CSCs)	IL-6, IL-8, VEGF, MMPs, M-CSF	[81]
Endothelial cells (ECs)	VEGF, ANG-1, PDGF, EGF	[82]
Mesenchymal stem cells (MSCs))	IL-4, IL-10, RANTES (CCL5), MCP-1 (CCL2), MCP-3 (CCL7), MIG (CXCL9), IP-10 (CXCL10)	[71,83]

VEGF expression is increased during hypoxia, when the concentration of HIF-1α, which has an affinity for the promoter of this gene, increases [84]. Within endothelial cells, there are receptors specific for VEGF, namely VEGF1 and VEGF2, with VEGF2 appearing to be of predominant importance. After VEGF binds to its receptor on the surface of the endothelial cells, messenger proteins are activated, which transmit the signal to the nucleus of the endothelial cell. The nuclear signal stimulates a group of genes to produce products necessary for the growth of new endothelial cells. Following the activation of endothelial cells by VEGF, matrix metalloproteinases (MMPs) are produced, which are responsible for the breakdown of the extracellular matrix. The matrix is responsible for the migration of endothelial cells, which begin to divide as they migrate into the surrounding tissues. They then transform into a network of blood vessels with the support of an adhesive factor, like integrin α or β. Newly formed blood vessels require stabilization or maturation with the help of angiotensin-1, -2 and their Tie-2 receptor.

Researchers indicated that angiogenic activators including VEGF exert a crucial role not only in the growth but in spread of tumors. The tissue levels of VEGF-C and VEGFR-2 are correlated with the progression and prognosis of the cancer [85,86,87,88].

In parallel with VEGF, platelet-derived growth factors (PDGFs) and their receptors (PDGFR) play a key role in stimulating angiogenesis to combat hypoxia in the tumor microenvironment. PDGFs act on tumor cells, vascular cells and stromal cells, modulating tumor growth, metastasis and the tumor microenvironment. PDGFs have been shown to be involved in the growth, invasion, angiogenesis and migration of many types of tumors, especially malignant tumors [89]. PDGF-β overexpression has been demonstrated in the capillaries of gliomas with endothelial cell hyperplasia, confirming its involvement in angiogenesis. Overexpression of PDGF-A and PDGF-B and their ligands is observed in gliomas. Also, these factors may impact astrocytoma development [90].

It has been shown that the invasive nature of breast cancer is strongly associated with PDGFR expression. PDGF is produced by platelets, but also by cancer cells. PDGFR binds to PDGFR in cancer cells and activates the receptor, triggering a series of biological processes [91]. Moreover, the transmembrane glycoprotein VI (GPVI) and its receptors for collagen and fibrin stimulate epithelial–mesenchymal transition (EMT) by stimulating platelet-derived growth factor (PDGF) secretion. GPVI is exclusively expressed by platelets and megakaryocytes, and this is associated with platelet activation. Interaction between GPVI and galectin-3 on the surface of colon cancer cells can cause platelet activation and subsequent PDGF release. PDGF then stimulates COX2 activation and the release of prostaglandin E2 from cancer cells by interacting with PDGFR. The PDGF/PDGFR signaling pathway plays an important role in tumor progression and metastasis [92]. Another study showed that PDGF and TGF signaling pathways are linked and promote epithelial–mesenchymal transition (EMT). The TGFβ/Smad signaling pathway has been recognized as a factor in the proliferation of malignant glioma cells, as it triggers PDGF autocrine and paracrine signaling [93,94]. The platelet-derived growth factor receptor alpha (PDGFRα) is an important factor influencing growth, angiogenesis and metastasis. The interaction of the PDGFR pathway with other signaling pathways may increase tumor growth and reduce the sensitivity of tumor cells to therapy. A relationship has also been observed between increased PDGFRα expression and wild-type RAS status in tumor cells and stromal adenocarcinoma cells, clearly suggesting that this can contribute to resistance to anti-EGFR therapy [95].

It has also been shown that PDGF-DD binds to PDGFR-β and activates the Notch1/Twist1 pathway in colorectal cancer. This results in increased tumor cell proliferation, angiogenesis and mesenchymal transition. Overexpression of PDGF-DD in colorectal cancer tissues modulates MMP-9 activity, which promotes tumor growth and migration [96]. Matrix metalloproteinases (MMPs) contribute to the invasion and metastasis of cancer cells by degrading the extracellular matrix (ECM) on the surface of cells. Overexpression of MMP 9 and MMP 2 is observed during development of gliomas, but they also play major roles in ECM degradation [97].

Expression of MMP genes is stimulated by pro-inflammatory cytokines like TNF-α, IL-1β, and IL-6. Activity of MMP is regulated by metalloproteinase tissue inhibitors (TIMPs), which is important for regulating degradation of ECM protein [98,99,100].

Interferon-γ (IFN-γ) is a pleiotropic cytokine secreted by multiple immune cell types, including CD8^+^ and CD4^+^ T cells, γδ T cells, natural killer (NK) cells, natural killer T (NKT) cells, B cells, macrophages, monocytes, and dendritic cells. IFN-γ binds to the IFN-γR1/2 receptor complex, activating the Janus kinase (JAK)–signal transducer and activator of transcription (STAT) pathway to regulate diverse cellular functions such as immune modulation, leukocyte trafficking, cell proliferation, apoptosis, antimicrobial defense, and both anti- and pro-tumor activities [101]. On one hand, it acts as a cytotoxic cytokine—working in concert with granzyme B and perforin to induce apoptosis in tumor cells. On the other hand, it promotes the expression of immune checkpoint molecules and indoleamine 2,3-dioxygenase (IDO), thereby enhancing immunosuppressive mechanisms [102].

IL-9 directly affects tumor cells in solid cancers. In colon cancer, IL-9 stimulates the proliferation of malignant epithelial cells by upregulating c-MYC and cyclin D1 expression in RKO and Caco-2 cell lines in vitro [103]. In lung cancer, IL-9 can promote the migration of A549 cells through an ICAM/LFA-1– and/or VCAM-1/Integrin-β–dependent mechanism [104]. Although in vitro studies show that IL-9 has minimal effects on proliferation, it enhances the expression of vascular endothelial growth factor (VEGF) and increases microvascular density, thereby promoting tumor growth via enhanced vascularization [105]. Furthermore, IL-9–producing Th9 and Th17 cells have been reported to drive metastasis and epithelial–mesenchymal transition (EMT) in both human and murine lung cancer cells by upregulating MMP-3, MMP-13, and other genes involved in angiogenesis and cell adhesion. Importantly, although Th9 and Th17 cells act cooperatively to promote metastasis, Th9 cells function differently from Th17 cells. Th9 cells specifically promote chemokine-mediated inflammation and angiogenesis, supporting tumor cell survival within the tumor microenvironment (TME) [106].

With the foundational concepts of propolis’s chemical nature, the challenges of carcinogenesis, and the pivotal roles of hypoxia and cytokines now established, the core of this review can be addressed. The following section will synthesize these elements, presenting a detailed analysis of the scientific evidence demonstrating how the bioactive compounds in propolis modulate the hypoxic response and the cytokine network within the tumor microenvironment.

## 5. The Effect of Different Types of Propolis and Its Compounds on the Tumor Microenvironment and Cytokine Release

The anticancer activity of propolis arises from a diverse array of specific, highly potent bioactive compounds rather than from a single, uniform mechanism [6,107]. This chapter presents a detailed mechanism-based analysis of the action of the two main bioactive components—Caffeic Acid Phenethyl Ester (CAPE) from European-type propolis and artepillin C from Brazilian green propolis—which exert their effects by directly targeting key signaling pathways within the tumor microenvironment [10,11,108]. While propolis contains a diverse array of flavonoids and phenolic acids, CAPE and artepillin C serve as prominent and multitarget effectors in modulating overlapping but distinct aspects of the HIF-1α, NF-κB, and STAT3 signaling network [6,109]. The following analysis will dissect their specific roles in these processes and their synergistic contributions to tumor microenvironment modulation. A synthesized overview of these targeted interactions is presented in Table 3, which links the principal molecular effectors of propolis to their respective signaling pathways and functional consequences within the TME. In propolis extracts from various geographical regions, CAPE and chrysin are among the most frequently identified bioactive compounds, both of which have been associated with anticancer activity. A central mechanism of tumor adaptation to hypoxic conditions is the stabilization and activation of hypoxia-inducible factor 1α (HIF-1α), which governs the expression of pro-angiogenic and pro-survival genes. Active constituents of propolis directly interfere with this critical pathway. Evidence demonstrates that CAPE and artepillin C inhibit the nuclear accumulation of HIF-1α and promote its degradation even under hypoxic conditions. Consequently, the transcription of HIF-1α target genes is downregulated, including that of Vascular Endothelial Growth Factor (VEGF), which is pivotal for angiogenesis. This effect has been confirmed in in vitro models, where extracts of Brazilian and Chilean propolis significantly reduced VEGF secretion by tumor cells, thereby inhibiting the formation of new blood vessels [110,111,112]. In Brazilian propolis, its main component artepillin C serves as the structural counterpart of CAPE. CAPE and artepillin C have been shown to bind mortalin–p53 complexes, thereby activating p53 and inducing growth arrest in human fibrosarcoma (HT1080), lung cancer (A549), and osteosarcoma (U2OS) cells [113]. Chronic inflammation within the TME is driven by positive feedback loops in which the transcription factors NF-κB and STAT3 play a key role. Propolis, and particularly CAPE, is a well-documented, potent, and specific inhibitor of NF-κB activation. The mechanism involves blocking the phosphorylation and degradation of its inhibitor, IκBα, thus preventing the translocation of NF-κB to the nucleus. This suppression of NF-κB activity directly translates into reduced expression of pro-inflammatory cytokines such as TNF-α, IL-6, and IL-1β, which are fundamental for tumor progression, angiogenesis, and immunosuppression [108,114].

Direct immunomodulatory effects are defined by the direct molecular interaction of propolis’s bioactive constituents, principally Caffeic Acid Phenethyl Ester (CAPE) and artepillin C, with intracellular signaling cascades within both cancer and immune cells. The primary example of this is the potent and specific inhibition of the NF-κB signaling pathway. By preventing the phosphorylation and subsequent degradation of its inhibitor, IκBα, CAPE directly abrogates the nuclear translocation of active NF-κB dimers in cells such as tumor-associated macrophages (TAMs) and cancer cells [6,10,108,114,115]. This action fundamentally halts the transcription of a suite of genes encoding key pro-inflammatory cytokines (e.g., TNF-α, IL-6, IL-1β) at their source, thereby directly reshaping the TME’s cytokine architecture [108,116].

Conversely, indirect immunomodulatory effects manifest as downstream consequences of propolis activity on the broader TME architecture, which subsequently modulates the immune landscape [6,107,117]. The potent anti-angiogenic action, mediated principally by the inhibition of the HIF-1α/VEGF pathway, serves as a paradigm of such an indirect mechanism. By suppressing VEGF production, propolis limits tumor neovascularization and may reduce tumor hypoxia [108,110,112]. These vascular modifications are suggested, in preclinical and molecular studies, to contribute to the normalization of tumor vasculature, producing a microenvironment potentially less conducive to the recruitment, survival, and immunosuppressive function of cell populations such as M2-polarized tumor-associated macrophages and myeloid-derived suppressor cells (MDSCs) [12,117]. As a result, this structural remodeling of the TME could facilitate a more effective anti-tumor immune response, as evidenced by enhanced infiltration and activity of effector lymphocytes in experimental models. This multitargeted approach underscores the unique therapeutic potential of propolis to disrupt the complex pro-tumorigenic network from multiple angles [107,117].

**Figure 1:**
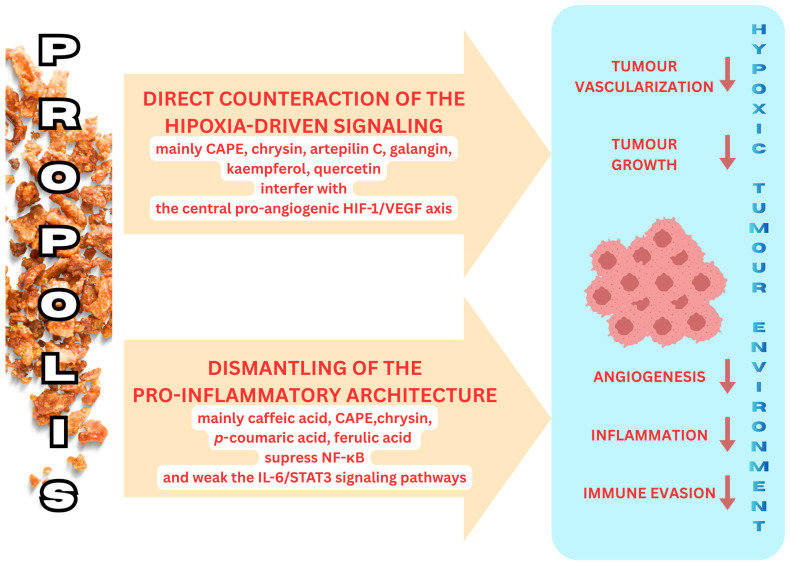
The effect of propolis and their components on signaling pathways in the tumor microenvironment.

Furthermore, by inhibiting IL-6 production, propolis indirectly weakens the pro-tumoral IL-6/JAK/STAT3 signaling pathway. Hyperactivation of the STAT3 pathway is correlated with an aggressive tumor phenotype and has been implicated in cancer cell survival and proliferation. The ability of propolis to simultaneously target the HIF-1α, NF-κB, and STAT3 pathways underscores its unique potential to dismantle the signaling network that sustains the hypoxic and pro-inflammatory tumor microenvironment [118,119]. This multi-level molecular intervention translates into a profound modulation of the TME’s cellular ecosystem. Propolis acts upon key cellular players, including cancer-associated fibroblasts (CAFs), tumor-associated macrophages (TAMs), and endothelial cells, to counteract tumor-promoting processes such as metastasis, immunosuppression, and angiogenesis. This complex interplay between propolis and the cellular components of the TME is visually summarized in Figure 1. The anticancer effects are observed across different types of propolis. Studies investigating the effects of ethanolic extracts of Polish propolis on cancer cell lines have demonstrated apoptosis-inducing activity in cervical cancer HeLa cells, with growth-inhibitory and pro-apoptotic effects occurring in a dose-dependent manner. Similarly, ethanolic extracts of propolis from south-eastern Poland inhibited proliferation in malignant melanoma (Me45) and colorectal cancer (HCT116) cells [120]. An ethanolic extract of brown propolis from Ceará—whose activity is influenced by regional environmental conditions—was found to inhibit approximately 75% of proliferation in human cancer cell lines, including prostate cancer (PC3), colon cancer (HCT116), and leukemia (HL60). However, this extract was non-selective, as it also suppressed the growth of normal cells. In contrast, a hexane extract inhibited cancer cell growth by 50–100%, demonstrating low cytotoxicity toward normal cells while maintaining high activity and selectivity against PC3 and HL60 cells [121]. Delving into the molecular mechanisms of individual components, chrysin participates in multiple signaling pathways that inhibit cancer cell proliferation. Moreover, it suppresses NF-κB and inhibitor of apoptosis proteins (IAPs), resulting in caspase-3 activation and subsequent apoptosis [1]. Caffeic acid and its ester have also been reported to induce S-phase cell cycle arrest in MDA-MB-231 breast cancer cells in a time- and dose-dependent manner, whereas CAPE reduces the proportion of cells in the G0/G1 phase [122]. Another research team has demonstrated the anti-tumor effects of CAPE on the same cell line, MDA-MB-231. Cells exposed to an inflammatory microenvironment induced by LPS were analyzed for changes in inflammatory mediators as well as key factors involved in glycolysis and lipid metabolism. CAPE treatment clearly suppressed cell proliferation, migration, invasion, and angiogenesis while also reducing mitochondrial membrane potential in LPS-stimulated MDA-MB-231 cells. Compared to the LPS-only group, CAPE reduced the expression of several pro-inflammatory mediators, including Toll-like receptor 4 (TLR4), tumor necrosis factor-alpha (TNF-α), NF-kappa-B inhibitor alpha (IκBα), IL-1β, IL-6, and interleukin-1 receptor-associated kinase 4 (IRAK4). Furthermore, CAPE significantly downregulated glucose transporters (GLUT1 and GLUT3) and major glycolytic enzymes—hexokinase 2 (HK2), phosphofructokinase (PFK), pyruvate kinase M2 (PKM2), and lactate dehydrogenase A (LDHA). It also reduced the expression of lipid metabolism-related proteins, including acetyl-CoA carboxylase (ACC), fatty acid synthase (FASN), and the fatty acid transporter CD36 [123].

CAPE combined with paclitaxel increased apoptosis and reduced migration in ovarian cancer OV7, HTB76, CRL1572 cell lines [124]. CAPE and its derivatives induced S and G2/M phase cell cycle arrest, modulated ROS levels, and activated autophagy signaling mTOR–ULK1–p62–LC3 in two oral squamous cell carcinoma cell lines, SAS and OECM-1 [125]. CAPE reduced viability, invasion and β-catenin signaling, AKT/GSK3β phosphorylation in gastric cancer cells [126].

In addition, CAPE inhibits ribosomal protein S6 kinase beta-1, a downstream effector of the PI3K/AKT pathway involved in protein synthesis, leading to suppression of proliferation in prostate cancer cell lines LNCaP, DU-145, and PC-3 [127].

The stabilization of HIF-1α is the cornerstone of the cellular response to low oxygen, coordinating a profound transcriptional shift that promotes tumor survival, metabolic reprogramming, and metastasis [19].

Crucially, propolis constituents modulate the HIF-1 pathway; CAPE can reduce HIF-1/VEGF signaling under hypoxia in retinal pigment epithelial (RPE) cells, but in other contexts stabilizes HIF-1α by inhibiting prolyl hydroxylase domain (PHD) enzymes [110,128,129]. A primary and direct downstream effect of HIF-1α activation is the potent transcriptional induction of VEGF, the key signaling protein for angiogenesis [130,131]. The link between propolis, hypoxia, and VEGF has been explicitly demonstrated. For instance, the ethanolic extract of Brazilian green propolis and its main component, artepillin C, significantly reduced IL-6 and VEGF levels under both hypoxic and normoxic conditions in the astrocyte cell line CCF-STTG1, mimicking the brain-tumor TME [132]. This indicates that propolis can directly counteract the strong angiogenic signal initiated by a low-oxygen environment. Furthermore, a Chinese red propolis blocked these processes in VEGF-stimulated human umbilical vein endothelial cells (HUVECs), indicating that propolis can act both upstream (by reducing VEGF production) and downstream (by inhibiting cellular responses to VEGF). This dual action makes propolis a robust natural agent for normalizing tumor vasculature and restraining tumor growth [133]. Ahn M.R. et al. found a correlation between antiangiogenic and antioxidant activities in various propolis components. Compounds such as artepillin C, caffeic acid phenethyl ester, galangin, kaempferol, and quercetin exhibited both strong antiangiogenic and antioxidant activities [134]. In turn Cuevas A. et al. demonstrated that the ethanolic extract of Chilean propolis can modulate in vitro angiogenesis in part by affecting the HIF-1α and ERK1/2 signaling pathways, with a mechanism involving miR-19b [112].

The hypoxic tumor microenvironment is inherently pro-angiogenic and pro-inflammatory. Key mediators such as TNF-α, IL-6, and IL-1β drive invasion, immunosuppression, and metastasis, with IL-6 notably activating the JAK/STAT3 pathway [36,38,135,136]. Hypoxia itself promotes cytokine production, and IL-6 can further amplify the hypoxic response via STAT3 and HIF-1α stabilization, forming a detrimental positive feedback loop that exacerbates tumor progression [137].

Frión-Herrera Y. have shown that Cuban propolis treatment of M2-like macrophages resulted in reduced mRNA expression of proinflammatory factors, including IL-8, IL-10, CCL2, and VEGF. Moreover, both Cuban propolis and its main component, nemorosone, significantly downregulated the activity of MMP-9, an enzyme released by M2-like macrophages that contributes to cancer cell infiltration and invasiveness. This effectively shifted the cytokine profile toward a state less supportive of tumors [138]. Propolis impedes angiogenesis, a process essential for tumor progression and metastasis. Propolis also exhibits anti-inflammatory activity by downregulating mediators such as tumor necrosis factor-α (TNF-α), cyclooxygenases (COX-1/2), inducible nitric oxide synthase (iNOS), lipoxygenases (LOX), prostaglandins (PGs), and interleukin-1β (IL-1β) [107]. Furthermore, in PC-3 cells, CAPE inhibits AKT/mTOR signaling and proliferation [139]. These findings indicate that propolis contains compounds capable of modulating the HIF-1/VEGF pathway, with the direction of effect dependent on the specific compound and cellular system [128]. By reducing IL-6 in hypoxia, the ethanolic extract of Brazilian propolis (EEP-B) may weaken the IL-6→STAT3→HIF-1α loop, thereby dampening pro-inflammatory and pro-angiogenic signaling within the TME, which could potentially limit tumor progression [36,132,137]. The anticancer effects of different types of Brazilian propolis are mediated through multiple mechanisms, including induction of apoptosis with concomitant reduction of cancer stem cells, cell cycle arrest, modulation of oncogenic signaling pathways, inhibition of tumor cell proliferation, suppression of metastasis and angiogenesis, and anti-inflammatory activity through modulation of the tumor microenvironment, particularly macrophage activation and polarization. Brazilian propolis compounds also mitigate chemotherapy-associated side effects and enhance cancer cell sensitivity to chemotherapy by inhibiting NF-κB activity [140]. Furthermore, it exerts pro-apoptotic effects through the activation of BAX, caspase-3, and cytochrome c [141]. Further supporting this, Li J. et al. [142] demonstrated that Chinese poplar propolis exhibits antitumor effects against human breast cancer MDA-MB-231 cells in an inflammatory microenvironment stimulated by lipopolysaccharide (LPS). Propolis significantly inhibited MDA-MB-231 cell proliferation, migration, invasion, colony formation, and angiogenesis. Concurrently, proinflammatory mediators, including tumor necrosis factor-alpha (TNF-a), interleukin (IL)-1β, and IL-6, as well as NLRP3 inflammasome activation, were reduced following propolis treatment compared with the LPS group [142]. One key mechanism behind these effects is the inhibition of the NF-κB pathway, which critically regulates inflammatory responses and can be modulated by hypoxia [143]. Notably, CAPE, a prominent propolis constituent, acts as a strong, specific inhibitor of NF-κB activation [114]. Moreover, beyond modulating soluble mediators, propolis directly influences immune cells within the TME. The predominant polyphenols in ethanolic extract of propolis—chrysin, caffeic acid, *p*-coumaric acid, and ferulic acid—reduce the viability of human tongue squamous cell carcinoma (CAL-27) cells by inhibiting DNA biosynthesis and inducing apoptosis via p53 activation. These polyphenols also decrease intracellular proline levels by promoting its mitochondrial degradation through proline dehydrogenase/oxidase (PRODH/POX), thereby triggering PRODH/POX-dependent apoptosis [144]. In another study, ethanolic, ethanol–hexane, and hexane extracts of Polish propolis demonstrated cytotoxicity against tongue cancer SCC-25 cells (CRL-1628), while exhibiting limited toxicity toward normal mucosal cells and concomitant anti-inflammatory activity [145]. Ethanolic extract of Polish propolis and its component quercetin modulate the tumor microenvironment in vitro, primarily by altering cytokine levels produced by astrocytes of the CCF-STTG1 cell line [146].

However, the therapeutic potential can vary significantly depending on the propolis origin. Studies of three propolis extracts—European, American, and Asian poplar—revealed moderate cytotoxicity against the osteosarcoma cell line MG63 and the leukemic cell lines HL60 and THP-1. To assess cancer cell specificity, the study also included a mouse connective tissue cell line and primary human mesenchymal stem cells (hMSCs), which are commonly used to evaluate biomaterial cytotoxicity and drug selectivity. Notably, higher cytotoxicity was observed in these non-cancerous cells. These findings highlight the variability in cytotoxic potential among different propolis types and extracts across studies [147].

**Figure 2:**
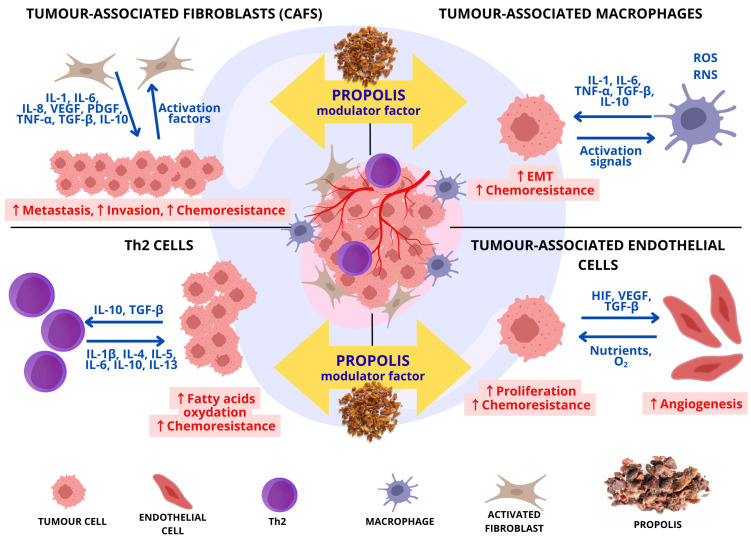
The effect of propolis on immunomodulation within the tumor microenvironment (based on [160]). Propolis acts as a modulator, interfering with the pro-tumorigenic signaling network established between cancer cells and key stromal components, including cancer-associated fibroblasts (CAFs), tumor-associated macrophages (TAMs), T helper 2 (Th2) cells, and tumor-associated endothelial cells. This intervention counteracts processes such as angiogenesis, metastasis, epithelial–mesenchymal transition (EMT), invasion, and chemoresistance. Abbreviations: EMT, epithelial–mesenchymal transition; HIF, hypoxia-inducible factor; IL, interleukin; O_2_, oxygen; PDGF, platelet-derived growth factor; RNS, reactive nitrogen species; ROS, reactive oxygen species; TGF-β, transforming growth factor-beta; Th2, T helper 2 cell; TNF-α, tumor necrosis factor-alpha; VEGF, vascular endothelial growth factor.

Due to its low water solubility, propolis can be delivered through nanoparticles or encapsulation, which may enhance its chemopreventive effects [148,149,150]. Amin A.A. et al. demonstrated that propolis nanocapsules exhibited high thermal stability and cytotoxic effects against three human cancer cell lines (PC3, MCF7, and HePG2) [149]. Tumor hypoxia is a significant challenge, promoting resistance to both radio- and chemotherapy [151]. Radiotherapy’s efficacy is diminished under low oxygen due to its dependence on reactive oxygen species generation. Additionally, hypoxic regions are often poorly vascularized, limiting the accessibility of cytostatics, and contain slower-proliferating cells that are less sensitive to many conventional drugs [152]. In this context, numerous studies have confirmed that propolis is safe and well tolerated, supporting its use as an adjuvant in various conditions. It can potentiate the effects of standard chemotherapy or radiotherapy while simultaneously reducing their adverse side effects. Selected propolis constituents, such as the ethanolic extract of Brazilian propolis (EEP-B), have been shown to modulate TME-relevant signals by reducing VEGF and IL-6 secretion in CCF-STTG1 astrocytes even under hypoxia [132], while CAPE acts as a strong, specific inhibitor of NF-κB activation [114]. However, direct evidence for radio- or chemosensitization of tumors by propolis specifically under hypoxic conditions remains limited, highlighting the need for dedicated studies in appropriate hypoxic models. The bioactive components of propolis targeting key signaling pathways in the tumor microenvironment are presented in Table 3 and Figure 1. Figure 2 illustrates the immunomodulatory effect of propolis on the tumor microenvironment.

### Limitations of Current Research and Future Directives

While the preclinical evidence supporting the anticancer potential of propolis is compelling, a critical evaluation reveals significant methodological limitations that must be addressed to facilitate its translation into clinical practice. These challenges temper the direct applicability of many published findings and necessitate a more rigorous and standardized approach in future research.

The profound chemical heterogeneity of propolis, dictated by its phytogeographical origin, seasonal variations, and bee species, presents a formidable methodological challenge [153]. Extracts from different regions possess distinct chemotypes (e.g., European poplar-type rich in CAPE vs. Brazilian green-type rich in artepillin C), making direct cross-study comparisons problematic [154,155,156].

Many studies fail to provide adequate chemical characterization (e.g., via HPLC-MS) of the extracts used [145,155].

This lack of standardization undermines the reproducibility of findings and obscures the precise identification of the bioactive constituents responsible for the observed effects. Future research must prioritize the use of chemically standardized, well-characterized propolis extracts to ensure data reliability and comparability [157].

A significant translational gap persists between preclinical findings, predominantly derived from simplified in vitro 2D cell culture models, and the complex, multifactorial reality of the in vivo tumor microenvironment (TME) [158]. Standard monocultures fail to recapitulate the three-dimensional architecture, cell–cell interactions (e.g., with stromal and immune cells), physiological drug delivery barriers, and hypoxic gradients characteristic of solid tumors. Consequently, while propolis may demonstrate potent cytotoxicity in a Petri dish, its ability to reach the tumor core at therapeutic concentrations and exert its immunomodulatory effects in situ remains largely unproven. A shift towards more sophisticated models, such as 3D organoids, co-culture systems, and robust in vivo animal studies, is imperative to validate these preliminary findings.

The current body of literature is marked by a conspicuous absence of standardized dosing protocols and a lack of data on the pharmacokinetic and pharmacodynamic properties of key propolis constituents [1,159]. Studies often utilize a wide range of concentrations in vitro and dosages in vivo without a clear rationale or correlation to achievable physiological levels. Furthermore, due to the poor water solubility of many active polyphenols, their bioavailability is a major concern. Without standardized, well-characterized formulations and corresponding dose–response studies, establishing a clear therapeutic window and translating preclinical data into safe and effective clinical protocols is impossible. Future work must focus on developing stable formulations with enhanced bioavailability and on conducting rigorous pharmacokinetic studies to guide rational dose selection.

## 6. Conclusions and Future Perspective

This review consolidates the evidence demonstrating that propolis exerts a significant immunomodulatory and anticancer effect within the hypoxic tumor microenvironment. Our analysis reveals that its potential stems from a powerful dual mechanism of action: (1) Direct counteraction of hypoxia-driven signaling: propolis constituents, especially CAPE presenting in the European Brown Type of propolis and artepillin C presenting in Brazilian green propolis, directly interfere with the central pro-angiogenic HIF-1/VEGF pathway, thereby limiting tumor vascularization and growth. (2) Dismantling of the pro-inflammatory architecture: propolis effectively disrupts the detrimental feedback loops that sustain chronic inflammation in the TME, primarily by suppressing the NF-κB and weakening the IL-6/STAT3 signaling pathway.

This ability to simultaneously target angiogenesis and inflammation—two cornerstones of tumor progression and therapy resistance—makes propolis a particularly compelling candidate for adjuvant therapy. However, a critical evaluation of the existing literature reveals significant knowledge gaps and conflicting data that must be addressed to translate these promising preclinical findings into clinical practice.

A central challenge is the paradoxical role of specific propolis constituents in modulating the HIF-1α pathway. For instance, while this review highlights evidence of CAPE reducing HIF-1/VEGF signaling under hypoxia, other studies have demonstrated that CAPE can, in fact, stabilize HIF-1α by inhibiting the prolyl hydroxylase domain (PHD) enzymes that target it for degradation. This dichotomous role is likely context-dependent, potentially varying based on cell type, oxygen tension, and the specific molecular background of the tumor, yet the precise determinants of this switch remain a critical knowledge gap. Elucidating the conditions under which propolis compounds act as either pro- or anti-HIF-1α stabilizing agents is paramount.

Furthermore, a significant translational gap exists between in vitro findings and potential in vivo efficacy. The vast majority of studies, including those discussed herein, are performed on 2D cell culture models. These systems fail to recapitulate the complex architecture of the TME, including spatial heterogeneity, stromal interactions, and physiological drug delivery barriers. Consequently, while we demonstrate that propolis can modulate cytokine profiles in vitro, its ability to achieve therapeutically relevant concentrations within the poorly vascularized, hypoxic core of a solid tumor and exert these effects in situ remains unproven.

To translate the promising preclinical data into a viable clinical strategy, future research must follow a structured, mechanistically driven trajectory. We propose the following experimental and clinical directions: (1) Advanced Preclinical Validation in TME-Mimicking Models: Moving beyond conventional 2D models, it is imperative to employ patient-derived tumor organoids and humanized mouse models (e.g., immunodeficient mice reconstituted with a human immune system). These systems will allow for the validation of the in vivo efficacy of chemically standardized propolis extracts in modulating a complex, multi-cellular human TME. Key experimental endpoints should include multiparametric analysis of immune cell infiltration (via immunohistochemistry and flow cytometry), quantification of key cytokine profiles (e.g., IL-6, TNF-α) within the tumor tissue, and assessment of vascular normalization. (2) Clinical Application as an Adjuvant Therapy: Propolis holds its greatest clinical promise not as a monotherapy but as a potent adjuvant agent. Specifically, future research should focus on its application as a chemosensitizing or radiosensitizing agent, designed to counteract therapy resistance in hypoxic, inflammation-driven solid tumors. We propose a Phase I/II clinical trial framework where standardized propolis is administered in a neoadjuvant setting (i.e., prior to standard-of-care surgery, chemotherapy, or radiotherapy) in cancers such as locally advanced pancreatic or head and neck squamous cell carcinoma, where hypoxia and inflammation are known drivers of therapeutic failure. This approach would provide a critical window to obtain pre- and post-treatment tumor biopsies to directly assess mechanistic endpoints. (3) Biomarker-Driven Patient Stratification: A “one-size-fits-all” approach is destined for failure. The proposed clinical trial design must be intrinsically linked to biomarker discovery. Based on its primary mechanisms of action, we hypothesize that tumors with high pre-treatment expression of HIF-1α or a strong inflammatory signature (e.g., high nuclear NF-κB activation, elevated serum IL-6) would be most susceptible to propolis-mediated sensitization. Pre-treatment biopsies should be analyzed for these molecular signatures to stratify patients most likely to respond. This would enable a transition from a generalized application to a targeted, biomarker-driven therapeutic strategy, which is the hallmark of modern oncology.

In conclusion, the dual capacity of propolis to simultaneously dismantle hypoxia-driven angiogenesis via the HIF-1α pathway and suppress the pro-tumorigenic inflammatory architecture via the NF-κB and STAT3 pathways distinguishes it from many single-target agents. This positions propolis not as a standalone curative but as a compelling candidate for adjuvant therapy, with the strategic potential to overcome therapy resistance in hypoxic and inflamed solid tumors. The critical path forward requires a paradigm shift from broad characterization to rigorous, mechanistically driven validation in clinically relevant models. If successful, harnessing the multi-faceted properties of propolis offers a rational, nature-based strategy to enhance the efficacy of conventional cancer therapies and improve patient outcomes.

## Figures and Tables

**Table 1:** Selection criteria for papers in this review.

Inclusion Criteria	Exclusion Criteria
1. Original preclinical studies (in vitro, in vivo) or mechanistic reviews. 2. Studies directly investigating the effects of propolis or its specific constituents (e.g., CAPE, artepillin C) on cancer cells or animal models 3. Articles providing insights into the modulation of hypoxia-related pathways (e.g., HIF-1α, VEGF) or key inflammatory/immune signaling networks (e.g., NF-κB, STAT3, cytokine production) by propolis.	1. Studies published in languages other than English. 2. Case reports, conference abstracts, editorials, and author opinions 3. Publications not directly related to the oncological context or the immunomodulatory role of propolis. 4. Duplicate publications of the same study.

**Table 3:** Mechanistic Summary of Propolis Bioactive Components Targeting Key Signaling Pathways in the Tumor Microenvironment (TME).

Bioactive Component	Primary Molecular Target/Pathway	Detailed Mechanism of Action	Resulting Biological Outcome in the TME
Caffeic Acid Phenethyl Ester (CAPE)	NF-κB (Nuclear Factor kappa-light-chain-enhancer of activated B cells)	Inhibits IκBα phosphorylation and degradation, thereby preventing the nuclear translocation of the active p65/p50 NF-κB dimer.	Suppression of chronic inflammation; reduced transcription of pro-inflammatory cytokines (TNF-α, IL-1β, IL-6) and pro-angiogenic factors.
CAPE & artepillin C	HIF-1α (Hypoxia-Inducible Factor 1-alpha)	Inhibit nuclear accumulation and promote proteasomal degradation of the HIF-1α subunit, even under hypoxic conditions.	Downregulation of HIF-1α target genes, most notably Vascular Endothelial Growth Factor (VEGF), leading to potent inhibition of tumor angiogenesis.
Propolis Extract (via CAPE and other polyphenols)	STAT3 (Signal Transducer and Activator of Transcription 3)	Indirectly weakens the IL-6/JAK/STAT3 pathway by suppressing the upstream production of IL-6 (a primary STAT3 activator).	Disruption of the pro-tumorigenic feedback loop sustaining cell proliferation, survival, and therapy resistance; reduction of cancer stem cell (CSC) population expansion.

## Data Availability

No new data were created or analyzed in this study. Data sharing is not applicable to this article.

## Abbreviations

The following abbreviations are used in this manuscript:

GC–MS	gas chromatography–mass spectrometry
PDK1	pyruvate dehydrogenase kinase 1
IL	interleukin
IFN	interferon
TNF	tumor necrosis factor
TGF	transforming growth factor
CSF	colony-stimulating factor
TAM	Tumor-associated macrophage
MMP	metalloproteinase
VEGF	vascular endothelial growth factor
bFGF	basic fibroblast growth factor
hMSCs	human mesenchymal stem cells
CDK	cyclin-dependent kinase
PDGF	platelet-derived growth factors
CAPE	caffeic acid phenethyl ester
MCP-1	monocyte chemotactic protein-1
LPS	lipopolysaccharide
TAMs	tumor-associated macrophages
IDO	indoleamine 2,3-dioxygenase
